# Combined *rpo*B duplex PCR and *hsp65* PCR restriction fragment length polymorphism with capillary electrophoresis as an effective algorithm for identification of Mycobacterial species from clinical isolates

**DOI:** 10.1186/1471-2180-12-137

**Published:** 2012-07-08

**Authors:** Chen-Cheng Huang, Jiann-Hwa Chen, Shiau-Ting Hu, Chien-Shun Chiou, Wei-Chang Huang, Jeng-Yuan Hsu, Jang-Jih Lu, Gwan-Han Shen

**Affiliations:** 1 Department of internal medicine, Executive Yuan Department of health, Division of Respiratory and Critical Care Medicine, Taichung Hospital, Taichung, Taiwan; 2 Institute of Molecular Biology, National Chung Hsing University, Taichung, 402, Taiwan; 3 Institute of Microbiology and Immunology, National Yang-Ming University, Taipei, Taiwan; 4 The Central Region Laboratory, Centers for Disease Control, Department of Health, Taichung, 408, Taiwan; 5 Division of Respiratory and Critical Care Medicine, Department of Internal Medicine, Taichung Veterans General Hospital, Taichung, Taiwan; 6 Department of Laboratory Medicine, Linkou Chang-Gung Memorial Hospital, Taoyuan, Taiwan; 7 Department of Respiratory Therapy, College of Health Care, China Medical University, Taiwan; 8 Institute of Nursing Care, Hungeuang University, Taichung, Taiwan; 9 Graduate Institute of Clinical Medical Science, China Medical University, Taichung, Taiwan

**Keywords:** *rpoB duplex polymerase chain reaction*, *hsp65 restriction fragment length polymorphism analysis,Capillary electrophoresis*

## Abstract

**Background:**

Mycobacteria can be quickly and simply identified by PCR restriction-enzyme analysis (PRA), but misidentification can occur because of similarities in band sizes that are critical for discriminating among species. Capillary electrophoresis can provide computer-aided band discrimination. The aim of this research was to develop an algorithm for identifying mycobacteria by combined *rpo*B duplex PRA (DPRA) and *hsp65* PRA with capillary electrophoresis.

**Results:**

Three hundred and seventy-six acid-fast bacillus smear-positive BACTEC cultures, including 200 *Mycobacterium tuberculosis* complexes (MTC) and 176 non-tuberculous mycobacteria (NTM) were analyzed. With combined *hsp65* and *rpo*B DPRA, the accuracy rate was 100% (200 isolates) for the MTC and 91.4% (161 isolates) for the NTM. Among the discordant results (8.6%) for the NTM, one isolate of *Mycobacterial species* and an isolate of *M. flavescens* were found as new sub-types in *hsp65* PRA.

**Conclusions:**

This effective and novel identification algorithm using combined *rpo*B DPRA and *hsp65* PRA with capillary electrophoresis can rapidly identify mycobacteria and find new sub-types in *hsp65* PRA. In addition, it is complementary to 16 S rDNA sequencing.

## Background

Detection and identification of mycobacteria in clinical specimens is a key issue in the therapy of pulmonary diseases because misidentification can lead to inappropriate treatment. Traditionally, mycobacterial species are identified based on their growth rate, presence or absence of pigmentation, and using biochemical assays of the isolates recovered from specimens. The biochemical assays are time-consuming and labor-intensive, usually taking 1 to 2 months to complete, and assays for non-tuberculous mycobacteria (NTM) species can have poor reproducibility and provide ambiguous results [1,2].

By contrast, molecular identification, notably PCR-restriction enzyme analysis (PRA), is rapid and simple. The *hsp65* PRA method, developed by Telenti et al. in 1993, is a popular DNA-based method for mycobacteria identification [3]. Using *hsp65* PRA, Wong et al. [4] reported 100% sensitivity and specificity in identifying *Mycobacterium tuberculosis* complexes but only 74.5% sensitivity in identifying NTM species. This misidentification may occur because of similarities in band sizes that are critical for species discrimination [3]. An additional contributing factor is a lack of knowledge of all existing PRA profiles, especially among species that are very heterogeneous, such as *M. gordonae*, *M. scrofulaceum*, and *M. terrae* complexes. Recently, capillary electrophoresis (CE) with computer analysis [5-9] has provided more precise band discrimination than analysis by the naked eye.

Previously, we developed an algorithm for mycobacterial species identification from acid-fast bacillus (AFB) smear-positive BACTEC tubes by combining the *rpo*B duplex PRA (DPRA) [10] and the key phenotypic characters of mycobacteria recovered from the tubes [11]. Using *rpo*B DPRA, we differentiated *Mycobacterium tuberculosis* complexes (MTC) from NTM with 235 base pair (bp) and 136 bp PCR amplicons in AFB smear-positive BACTEC cultures. The 136 bp *rpo*B duplex PCR amplicon was further digested with *Msp*I and *Hae*III (*rpo*B DPRA) to divide the NTM species into eight easily distinguishable groups (A–H) as described by Kim et al. [10]. Using two phenotypic characters (growth rate and photoreactivity on pigment production) and two simple biochemical assays (nitrate reduction test and Tween 80 hydrolysis test) [11], the mycobacterial species were identified. However, the sub-culture and biochemical tests for this algorithm took three weeks.

In the present study, we developed a rapid and effective algorithm for identification of mycobacteria by combined *rpo*B DPRA and *hsp65* PRA with CE.

## Results

### Mycobacteria identification

There were 376 AFB smear-positive BACTEC culture tubes (positive BACTEC cultures), including 200 MTC and 176 NTM-containing BACTEC cultures. A further 20 bacteria were MGIT positive but AFB culture smear negative, and these were classed as contaminated and excluded from subsequent evaluation.

**Figure 1:**
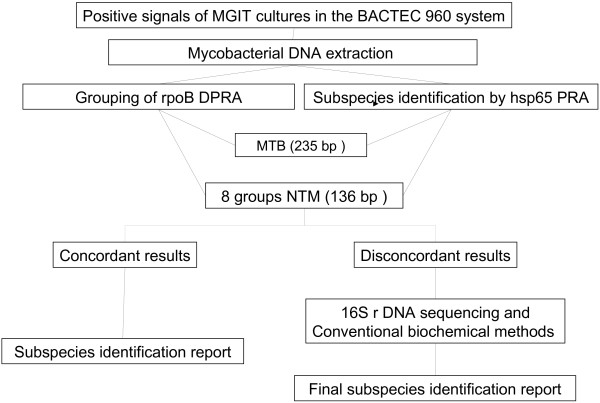
**An flow chart of the identification of Mcobacterial species from clinical specimens by combined *rpo *B duplex PCR(DCR) and *hsp *65 PCR-Restriction Fragment Length Polymorpgism analysis(PRA).**

By *rpo*B duplex PCR, all of the 200 MTC-containing BACTEC cultures and the 176 NTM-containing BACTEC cultures showed 235-bp and 136-bp PCR amplicons specific for MTC and NTM, respectively. The species were identified according to the flow chart shown in Figure 1.

### Concordant results from *rpo*B DPRA and *hsp65* PRA

Combining *rpo*B DPRA and *hsp65* PRA with computer-aided CE gave an accuracy rate of 100% (200/200) for MTC and 91.4% (161/176) for NTM (Table 1).

**Table 1:** **Comparison of *hsp65 *RFLP, *rpo *B RFLP patterns, 16 S rDNA sequences and conventional biochemical identification in 361 isolates with concordant results**

***rpo *B RFLP pattern**	***hsp65 *RFLP pattern**	**16 S rDNA sequence identification**	**Conventional biochemical**	**BACTEC culture number**	**Concordance rate**
			**identification**		
**T**	*M. tuberculosis type 1*	*M. tuberculosis*	*M. tuberculosis*	200	100%(200/200)
	NTM	NTM	NTM	161	91.4%(161/176)
**A**	*M. abscessus* type1	*M. abscessus*	*M. abscessus*	29	
**A**	*M. abscessus* type 2	*M. abscessus*	*M. abscessus*	41	
**A**	*M. fortuitum* type 1	*M. fortuitum*	*M. fortuitum*	33	
**A**	*M. fortuitum* type 2	*M. fortuitum*	*M. fortuitum*	2	
**A**	*M. peregrinum* type 1	*M. peregrinum*	M. fortuitum*	5	
**A**	*M. peregrinum* type 2	*M. peregrinum*	M. fortuitum*	8	
**A**	*M. peregrinum* type 3	*M. peregrinum*	M. fortuitum*	1	
**A**	*M. chelonae* type 1	*M. chelonae*	*M. chelonae*	1	
**A**	*M. mucogenicum* type 1	*M. mucogenicum*	*M. mucogenicum*	2	
**A**	*M. smegmatis* type 1	*M. smegmatis*	*M. smegmatis*	2	
**B**	*M. avium subsp. avium* type 2	*M. avium subsp. avium*	M. avium complex**	2	
**D**	*M. kansasii* type 1	*M. kansasii*	*M. kansasii*	6	
**D**	*M. kansasii* type 2	*M. kansasii*	*M. kansasii*	1	
**D**	*M. kansasii* type 6	*M. kansasii*	*M. kansasii*	1	
**D**	*M. triviale* type 1	*M. triviale*	*M. triviale*	1	
**F**	*M. malmoense* type 1	*M. malmoense*	*M. malmoense*	2	
**F**	*M. szulgai* type 1	*M. szulgai*	*M. szulgai*	1	
**F**	*M. interjectum type 1*	*M. interjectum*	*M. interjectum*	1	
**G**	*M. intracellulare* type 1	*M. intracellulare*	M. avium complex**	14	
**G**	*M. gordonae* type 1	*M. gordonae*	*M. gordonae*	6	
**G**	*M. gordonae* type 2	*M. gordonae*	*M. gordonae*	1	
**G**	*M. gordonae* type 5	*M. gordonae*	*M. gordonae*	1	
**Total**				361	

* *M. peregrinum* was identified as *M. fortuitum* by a conventional biochemical method.

**M. avium subsp. avium and M. intracellulare were identified as M. avium complex by a conventional biochemical method.

### Discordant results from *rpo*B DPRA and *hsp65* PRA

There were 15 isolates (8.6%) of NTM with discordant results with *rpo*B DPRA and *hsp65* PRA (Table 2). The two isolates, *Mycobacterial species* (A group) and *M. flavescens* (A group) identified by 16 S rDNA sequencing represented new patterns not available in the *hsp*65 PRA databases and might be new sub-types in *hsp65* PRA. For *Mycobacterial species,* 16 S rDNA sequencing did not confirm the identity of the isolate but conventional biochemical identification showed it was *M. mucogenicum.*

**Table 2:** **Fifteen isolates of NTM species with discordant results from*rpo*B RFLP,*hsp65*RFLP patterns, 16 S rDNA sequence and conventional biochemical identification**

***No***	***rpo *B RFLP pattern**	***hsp65 *RFLP pattern**	**16 S rDNA sequence**	**Conventional biochemical identification**
**1**	**A**	*Bst*EII : 242.8*, 214.0, 0	*M. flavescens*	*M. flavescens*
		*Hae*III: 130.9, 140, 90.4, 49.7, 41.5, 37.1		
**2**	**A**	*Bst*EII :456.3, 0, 0	*Mycobacterial species*	*M. mucogenicum*
		*Hae*III:192.6, 90.4, 82.0		
**3**	**D**	*M. scrofulaceum type 1*	*M. scrofulaceum*	*M. scrofulaceum*
**4**	**G**	*M. simiae* type 5	*M. simiae*	*M. simiae*
**5**	**G**	*M. simiae* type 5	*M. simiae*	*M. simiae*
**6**	**F**	*M. intracellulare* type 3	*M. intracellulare*	M. avium complex**
**7**	**F**	*M. gordonae* type 3	*M. gordonae*	*M. gordonae*
**8**	**F**	*M. gordonae* type 3	*M. gordonae*	*M. gordonae*
**9**	**F**	*M. gordonae* type 3	*M. gordonae*	*M. gordonae*
**10**	**F**	*M. gordonae* type 3	*M. gordonae*	*M. gordonae*
**11**	**F**	*M. gordonae* type 3	*M. gordonae*	*M. gordonae*
**12**	**F**	*M. gordonae* type 3	*M. gordonae*	*M. gordonae*
**13**	**F**	*M. gordonae* type 3	*M. gordonae*	*M. gordonae*
**14**	**F**	*M. gordonae* type 4	*M. gordonae*	*M. gordonae*
**15**	**F**	*M. gordonae* type 4	*M. gordonae*	*M. gordonae*

### Development of a species identification algorithm

The results in Tables 1 and 2 and the mycobacterial identification flow chart (Figure 1) were used to develop a species identification algorithm by combining *rpo*B duplex PCR [10] and *hsp65* PRA [3] using the most common 74 patterns of 40 species in Table 3. In this algorithm (Table 3), we added *M. gordonae* types 3 and 4 to the F group. The accuracy rate for NTM by combining the two methods could reach 96.6% (170/176).

**Table 3:** **A species identification algorithm by combining *rpo *B duplex PCR and *hsp65 *PCR-restriction fragment length polymorphism analysis**

	***rpo *B DPCR-RFLP**		***hsp *65 RFLP**		**Final species identification**
**Pattern**	**M*sp*I**	**H*ae*III**	**BstEII**	**Hae III**	
**A**	136	136	440 / 0 / 0	160 / 90 / 60 / 0	*M. vaccae* type 1
			440 / 0 / 0	160 / 85 / 55 / 0	*M. flavescens* type 3
			440 / 0 / 0	140 / 55 / 50 / 0	*M. flavescens* type 1
			440 / 0 / 0	130 / 115 / 70 / 60	*M. aurum* type 2
			320 / 130 / 0	200 / 70 / 60 / 55	*M. immunogenum* type 2
			320 / 130 / 0	200 / 60 / 55 / 50	*M. chelonae* type 1
			320 / 130 / 0	145 / 70 / 60 / 55	*M. immunogenum* type 1
			320 / 130 / 0	140 / 65 / 60 / 0	*M. mucogenicum* type 1
			320 / 115 / 0	185 / 145 / 0 / 0	*M. fallax* type 1
			320 / 115 / 0	170 / 140 / 0 / 0	*M. neoaurum* type 1
			320 / 115 / 0	145 / 65 / 60 / 0	*M. mucogenicum* type 2
			320 / 115 / 0	140 / 90 / 60 / 0	*M. mucogenicum* type 3
			235 / 210 / 0	200 / 70 / 60 / 50	*M. abscessus* type 2
			235 / 210 / 0	180 / 135 / 70 / 50	*M. thermoresistibile* type 1
			235 / 210 / 0	145 / 140 / 100 / 50	*M. peregrinum* type 1
			235 / 210 / 0	145 / 70 / 60 / 55	*M. abscessus* type 1
			235 / 210 / 0	140 / 125 / 100 / 50	*M. peregrinum* type 2
			235 / 210 / 0	140 / 125 / 60 / 50	*M. senegalense* type 3
			235 / 210 / 0	140 / 80 / 60 / 50	*M. phlei* type 1
			235 / 210 / 0	130 / 80 / 60 / 0	*M. celatum* type 1
			235 / 130 / 85	175 / 80 / 0 / 0	*M. aurum* type 1
			235 / 130 / 85	145 / 140 / 100 / 60	*M. peregrinum* type 3
			235 / 130 / 85	145 / 125 / 60 / 0	*M. smegmatis* type 1
			235 / 130 / 85	140 / 125 / 60 / 50	*M. senegalense* type 2
			235 / 120 / 85	180 / 140 / 50 / 0	*M. senegalense* type 4
			235 / 120 / 85	145 / 120 / 60 / 55	*M. fortuitum* type 1
			235 / 120 / 85	140 / 125 / 60 / 50	*M. senegalense* type 1
			235 / 120 / 85	140 / 120 / 60 / 55	*M. fortuitum* type 2
			235 / 120 / 85	135 / 90 / 85 / 0	*M. fortuitum* type 3
**B**	136	108,28	320 / 115 / 0	140 / 90 / 60 / 0	*M. chitae* type 1
			235 / 210 / 0	145 / 130 / 0 / 0	*M. avium* subsp. *avium* type 3
			235 / 210 / 0	130 / 105 / 60 / 0	*M. avium* subsp. *avium* type 2
			235 / 210 / 0	130 / 105 / 0 / 0	*M. avium* subsp. *avium* type 1
			235 / 210 / 0	130 / 105 / 0 / 0	*M. avium* subsp. *paratuberculosis* type 1
**C**	136	76,60	235 / 120 / 85	160 / 105 / 60 / 0	*M. xenopi* type 1
**D**	75,61 or	136	440 / 0 / 0	170 / 130 / 0 / 0	*M. triviale* type 1
	75,57,4		320 / 115 / 0	130 / 95 / 75 / 60	*M. kansasii* type 5
			235 / 210 / 0	190 / 105 / 80 / 0	*M. ulcerans* type 2
			(235 / 210 / 0	145 / 130 / 95 / 0	*M. scrofulaceum* type 1*)
			235 / 210 / 0	145 / 105 / 80 / 45/20	*M. marinum* type 1
			235 / 210 / 0	145 / 105 / 80 / 0	*M. ulcerans* type 1
			235 / 210 / 0	130 / 105 / 80 / 0	*M. kansasii* type 1
			235 / 130 / 85	140 / 105 / 70 / 0	*M. shimodei* type 1
			235 / 120 / 85	130 / 115 / 75 / 60	*M. kansasii* type 4
			235 / 130 / 85	130 / 105 / 70 / 0	*M. kansasii* type 6
			235 / 130 / 85	130 / 105 / 0 / 0	*M. kansasii* type 2
			235 / 130 / 85	130 / 95 / 70 / 0	*M. kansasii* type 3
**E**	75,61 or	108,28	440 / 0 / 0	145 / 130 / 0 / 0	*M. simiae* type 5
	75,57,4		320 / 115 / 0	185 / 140 / 0 / 0	*M. terrae* type 2
			320 / 115 / 0	180 / 130 / 0 / 0	*M. terrae* type 1
			320 / 115 / 0	145 / 130 / 0 / 0	*M. simiae* type 4
			320 / 115 / 0	140 / 90 / 60 / 0	*M. nonchromogenicum* type 2
			320 / 115 / 0	140 / 60 / 50 / 0	*M. terrae* type 3
			320 / 115 / 0	125 / 105 / 0 / 0	*M. genavense* type 1
			235 / 210 / 0	185 / 130 / 0 / 0	*M. simiae* type 1
			235 / 210 / 0	185 / 130 / 0 / 0	*M. genavense* type 2
			235 / 210 / 0	155 / 140 / 0 / 0	*M. simiae* type 2
			235 / 210 / 0	145 / 130 / 0 / 0	*M. simiae* type 6
			235 / 210 / 0	140 / 115 / 70 / 0	*M. terrae* type 4
			235 / 130 / 85	145 / 130 / 0 / 0	*M. simiae* type 3
			235 / 130 / 85	130 / 105 / 70 / 0	*M. gastri* type 1
			235 / 120 / 85	145 / 60 / 55 / 0	*M. nonchromogenicum* type 1
**F**	75,61 or	76,60	440 / 0 / 0	130 / 105 / 70 / 0	*M. szulgai* type 1
	75,57,4		(320 / 115 / 0	130 / 115 / 60 / 0	*M. gordonae* type 4*)
			240/210/0	130/110/0	*M. interjectum*
			(235 / 210 / 0	145 / 130 / 0 / 0	*M. intracellulare* type 3*)
			235 / 210 / 0	115 / 105 / 0 / 0	*M. asiaticum* type 1
			235 / 130 / 85	130 / 105 / 80 / 0	*M. celatum* type 2
			235 / 120 / 100	145 / 105 / 80 / 0	*M. malmoense* type 1
			235 / 210 / 0	145 / 105 / 80 / 0	*M. malmoense* type 2
			(235 / 120 / 100	130 / 115 / 0 / 0	*M. gordonae* type 3*)
**G**	75,61 or	76,32,28	(440 / 0 / 0	145 / 130 / 0 / 0	*M. simiae* type 5*)
	75,57,4		320 / 115 / 0	130 / 110 / 70 / 60	*M. gordonae* type 8
			320 / 115 / 0	130 / 115 / 60 / 0	*M. gordonae* type 4
			235 / 210 / 0	145 / 130 / 0 / 0	*M. intermedium* type 1
			235 / 210 / 0	145 / 130 / 0 / 0	*M. intracellulare* type 3
			235 / 210 / 0	140 / 105 / 80 / 0	*M. intracellulare* type 2
			235 / 210 / 0	130 / 115 / 0 / 0	*M. gordonae* type 5
			235 / 210 / 0	120 / 115 / 110 / 0	*M. intracellulare* type 4
			235 / 130 / 85	140 / 120 / 95 / 0	*M. gordonae* type 6
			235 / 120 / 100	160 / 115 / 60 / 0	*M. gordonae* type 9
			235 / 120 / 100	155 / 110 / 0 / 0	*M. gordonae* type 7
			235 / 120 / 100	145 / 130 / 60 / 0	*M. intracellulare* type 1
			235 / 120 / 100	130 / 115 / 0 / 0	*M. gordonae* type 3
			235 / 120 / 100	130 / 110 / 95 / 0	*M. gordonae* type 10
			235 / 120 / 85	160 / 115 / 60 / 0	*M. gordonae* type 1
			235 / 120 / 85	215 / 110 / 0 / 0	*M. gordonae* type 2
**H**	75,61 or	66,60,10	235 / 210 / 0	145 / 130 / 95 / 0	*M. scrofulaceum* type 1
	75,57,4		320 / 130 / 0	160 / 110 / 0 / 0	*M. haemophilum* type 1
**T**			235 / 120 / 85	150 / 130 / 70 / 0	*M. tuberculosis* type 1
**(235 bp)**					

(*)species from Table 2 were included in this algorithm.

## Discussion

Mycobacterial species identification is generally achieved by standard culture and biochemical methods [12]. Biochemical methods are labor-intensive and time-consuming, and not all are reproducible [2,13]. Recently, PRA and DPRA have been developed for molecular identification of mycobacterial species using different regions of *hsp65*, 16 S rDNA, 16 S-23 S rDNA spacer, *dnaJ*, and *rpo*B as an amplification target [3,14-17]. The most common method is *hsp65* PRA, and 74 patterns for 40 species are available in the PRASITE database (http://app.chuv.ch/prasite/index.html).

Previous studies [18,19] have reported that *hsp65* PRA is faster and more accurate for species identification than conventional (phenotypic or biochemical) testing. This is because more incorrect and ambiguous results are obtained with conventional methods. The results in our study (Tables 1 and 2) also support this finding. Incorrect and ambiguous results are caused by phenotypic homogeneity among different species and phenotypic variability within species [18]. With by *hsp65* PRA, some sub-species, such as *M. kansasii*, can be identified and rapid-growing mycobacterium can be divided into *M. abscessus* and *M.chelonae*, *M. fortuitum* and *M. smegmatis*[20], whereas these identifications are difficult with conventional methods [21]. As found in our study (Tables 1 and 2), *M. peregrinum* was identified as *M. fortuitum* and *M. avium subsp. avium* and *M. intracellulare* were both identified as *M. avium complex* by the conventional biochemical method.

However, *hsp65* PRA limitations have been reported in some articles [22,23]. Failure to identify or incorrect identification of the species may occur because of similarities in band sizes critical for discriminating species, including difficult to distinguish *M. tuberculosis* complex (*M. tuberculosis* and *M. bovis*) [22], and closely related sub-species such as *M. avium* or *M. gordonae*, because of sequence heterogeneity [22]. In addition, technical problems can also cause misinterpretation or incorrect identification [23]. Patterns in PRA profiles are complex and difficult to interpret with the naked eye, especially when more detailed sub-types are included [21].

This study combined *rpo*B DPRA and *hsp65* PRA to test both reference strains and clinical respiratory isolates. The mycobacterial identification flow chart (Figure 1) can identify species to the sub-species level, and final species identification can be obtained instantly with concordant results from the two PRA. *M. gordonae* has a highly variable gene sequence with 10 sub-types in *hsp65* PRA, and there are two groups (G and F) in *rpoB* DPRA. Most *M. gordonae* is in the G group, but *M. gordonae* types 3 and 4 by *hsp65* PRA are in the F group (Tables 1 and 2).

In addition, there were different *rpo*B DPRA results (Table 2) for *M. simaie* type 5 (G group but not E group), *M. scrofulaceum* type 1 (D group but not H group), and *M. intracellulare* type 3 (F group but not G group). The identities of all of these isolates were finally confirmed by 16 S rDNA sequencing. Variable numbers of restriction sites for *Hae*III in these species may derive from genetic sequence mutation. However, these species are included in the species identification algorithm even though they are uncommon isolates.

Using the mycobacteria identification flow chart (Figure 1) and algorithm (Table 3), *M. avium-intracellulare* complex (MAC) can be easily divided into *M. avium spp. avium* and *M. intracellulare* by both *rpo*B DPRA and *hsp65* PRA. By contrast, this was not possible with the conventional method. Using the results in Table 3, some NTM species with identical or similar *hsp65* PRA can be clearly grouped by *rpoB* DPRA (Table 4). Ambiguous results from *hsp65* PRA alone are easier to interpret with combined *rpo*B DPRA and *hsp65* PRA. However, *M. intermedium* type 1 and *M. intracellulare* type 3 with identical *hsp65* PRA and *rpo*B DPRA (G group) could not be differentiated further by this species identification algorithm and required 16 S rDNA sequencing for confirmation.

**Table 4:** **Species with identical or similar *hsp65 *PRA but different groups in *rpoB *DPRA**

***rpo *B Group**	**Species (type)**	***hsp *65 RFLP**	
		***Bst *EII**	***Hae *III**
A	*M. mucogenicum* type 3	320 / 115 / 0	140 / 90 / 60 / 0
B	*M. chitae* type 1	320 / 115 / 0	140 / 90 / 60 / 0
A	*M. mucogenicum* type 2	320 / 115 / 0	145 / 65 / 60 / 0
E	*M. terrae* type 3	320 / 115 / 0	140 / 60 / 50 / 0
A	*M. fallax* type 1	320 / 115 / 0	185 / 145 / 0 / 0
E	*M. terrae* type 2	320 / 115 / 0	185 / 140 / 0 / 0
A	*M. peregrinum* type 2	235 / 210 / 0	140 / 125 /100/50
H	*M. scrofulaceum* type 1	235 / 210 / 0	145 / 130 / 95 / 0
D	*M. kansasii* type 6	235 / 130 / 85	130 / 105 / 70 / 0
E	*M. gastri* type 1	235 / 130 / 85	130 / 105 / 70 / 0
F	*M. celatum* type 2	235 / 130 / 85	130 / 105 / 80 / 0
D	*M. kansasii* type 1	235 / 210 / 0	130 / 105 / 80 / 0
F	*M. malmoense* type 2	235 / 210 / 0	145 / 105 / 80 / 0
E	*M. simiae* type 6	235 / 210 / 0	145 / 130 / 0 / 0
G	*M. intermedium* type 1	235 / 210 / 0	145 / 130 / 0 / 0
G	*M. intracellulare* type 3	235 / 210 / 0	145 / 130 / 0 / 0
F	*M. interjectum*	240 / 210 / 0	130 / 110 / 0
G	*M. gordonae* type 5	235 / 210 / 0	130 / 115 / 0 / 0

Although 16 S rDNA sequencing is the standard method for mycobacterium species identification, it cannot differentiate some closely related rapid-growing mycobacterium species [24] or slow-growing *M. kansasii* and *M. gastri* that had identical 16 S rDNA sequences, but these can be differentiated by *hsp65* PRA and *rpo*B DPRA. There are some reports [6,25] of conflicting results from different methods for mycobacterial species identification, probably caused by a failure of one method to identify all test strains correctly. Combining methods for mycobacterial species identification can improve the accuracy rate, avoid ambiguous results, and save time.

Many CE-based studies [5-9] in PCR-RFLP analysis have investigated improving band size discrimination. In one study by Chang et al. [7], high-resolution CE gave more precise estimates of DNA fragment sizes than analysis by the naked eye, and CE could detect low molecular weight fragments (down to 12 bp). In our study, restriction fragments <50 bp were not selected for analysis to avoid confusion with primer and primer dimer bands. We found that more *hsp*65 fragment differences than *rpo*B fragment (data not shown) may explain the size differences with highly variable sequence for species identification but difficult interpretation in *hsp65* PRA.

Some sub-types of NTM species are relevant to clinical management, such as the *M. kansasii* and MAC. *M. kansasii* type 1 is the most common type associated with human disease [26-28] because of its high pathogenicity. However, *M. kansasii* types 3–7 are most often isolated from the environment and rarely from humans, and have no significant role in clinical management [26,27]. MAC can be divided into *M. avium subsp. avium* and *M. intracellulare* because drug sensitivity test and clinical outcomes are different between these two sub-types [29,30].

It is important to identify NTM to the sub-type level both for epidemiologic data and for differentiating potentially pathogenic sub-types [26,27]. Combined *rpo*B DPRA and *hsp65* PRA with capillary electrophoresis provides precise species identification and overcomes problems associated with discrimination by *hsp65* PRA band sizes. This combined method takes around 2–3 days to complete in the laboratory once clinical isolates have been received. However, the identification algorithm has some limitations. First, it could not discriminate *M. intermedium* type 1 from *M. intracellulare* type 3, and second, not every hospital laboratory will be equipped with the appropriate equipment for this method.

## Conclusion

In conclusion, the novel flow chart and identification algorithm obtained by combined *rpo*B DPRA and *hsp65* PRA with capillary electrophoresis can easily differentiate MTC from NTM and identify mycobacterial species to the sub-type level, which is helpful for clinical management. The results are complementary to 16 S rDNA sequencing, and the effective algorithm provides rapid and accurate mycobacterial species identification.

## Methods

### Mycobacterial isolates

Fourteen mycobacterial reference strains including one MTC and 13 NTM strains and 376 clinical respiratory specimens, including sputum, broncho-alveolar lavage, and aspirated secretion from endotracheal tubes, were collected from January to July 2007 from Taichung Veterans General Hospital (Taichung, Taiwan). The respiratory specimens were digested by a *N*-acetyl-l-cysteine-NaOH decontamination procedure, centrifugal concentration, and sputum dissolving agents [31]. The processed specimens or the concentrated specimens were inoculated into MGIT culture tubes and incubated in the BACTEC 960 instrument at 37°C until a positive signal appeared. Positive BACTEC cultures were smeared on glass slides and Kinyoun staining was used to screen for AFB [31]. Mycobacteria in the positive BACTEC cultures were isolated and identified by conventional methods [12,13] .

### Identification of mycobacterial isolates by conventional methods

All 376 isolates from cultures in the AFB smear-positive BACTEC tubes were streaked on Löwenstein-Jensen (LJ) slants and incubated at 37°C under 5% CO_2_. Colonies on the LJ slants were used for species identification by conventional culture and biochemical methods [12,13]. These methods included growth rates, photoreactivity for pigment production, morphology in microcolonies on LJ slants, and biochemical tests, including nitrate reduction, arylsulfatase, Tween 80 hydrolysis, urease, semiquantitative catalase, tolerance to 5% NaCl and niacin production.

### Genomic DNA extraction

Mycobacterial DNA was extracted from positive BACTEC cultures using a DTB specimen processing kit (Becton Dickinson, Franklin Lakes, NJ) according to the manufacturer’s instructions [11].

### *rpoB DPCR and rpoB DPRA*

The r*po*B DPCR was performed using genomic DNA as template and primer pairs Tbc1 (5’-CGTACGGTCGGCGAGCTGATCCAA-3’)-TbcR5 (5’-CCACCAGTCGGCGCTTGTGGGTCAA-3’) and M5 (5’-GGAGCGGATGACCACCCAGGACGTC-3’)-RM3 (5’-CAGCGGGTT GTTCTGGTCCATGAAC-3’) as described by Kim et al. [10]. A 235 bp DNA PCR amplicon from MTC and a 136 bp DNA PCR amplicon from NTM were specifically amplified [10], and these two amplification products were analyzed by electrophoresis on a 2% agarose gel (Seakem LE agarose, Cambrex, East Rutherford, NJ).

For *rpo*B DPRA, the 136-bp DNA PCR amplicon was further digested with *Msp*I and *Hae*III after DPRA, and analyzed by electrophoresis on a 3% agarose gel (NuSieve 3:1 agarose, Cambrex) or CE (eGene). The *rpo*B restriction fragment length polymorphism (RFLP) patterns were compared to eight groups described by Kim et al. [10]. Eight NTM reference strains (*M. abscessus* ATCC 19977, *M. avium subsp. avium* ATCC 25291, *M. kansasii* ATCC 12479, *M. terrae* ATCC 15755, *M. szulgai* ATCC 29716, *M. intracellulare* ATCC 13950, *M. scrofulaceum* ATCC 19981, *M. xenopi* ATCC 19250) from each *rpo*B group (A-H) were subjected to *rpo*B DPRA by CE (eGene).

### *hsp65 PCR and hsp65PRA*

The *hsp65* PCR was performed using genomic DNA as template and primer Tb11(5’-ACC AAC GAT GGT GTG TCC-3’) and Tb12 (5’-CTT GTC GAA CCG CAT ACC CT-3’) as described by Telenti et al. [3]. A 439-bp DNA *hsp*65 PCR amplicon was specifically amplified from the extracted DNA, and the amplification product was analyzed by electrophoresis on a 2% agarose gel (Seakem LE agarose, Cambrex).

For *hsp65* PRA, the 439-bp DNA *hsp65* PCR amplicon was further digested with *BstE*II and *Hae*III after completing *hsp65* PCR, and analyzed by electrophoresis on a 3% agarose gel (NuSieve 3:1 agarose, Cambrex) or by CE (eGene). The sizes of the restriction fragment by *hsp65* PRA were compared to those reported on the PRASITE database (http://app.chuv.ch/prasite/index.html). Thirteen ATCC NTM reference strains and one MTC reference strain were subjected to *hsp65* PRA by CE (eGene).

### eGene CE

The restriction fragment sizes were estimated by the naked eye or by eGene CE according to the manufacturer’s instructions using size markers (100 bp to 3 kb) and alignment markers (15 bp to 3 kb).

### Mycobacterial identification flow chart

The mycobacterial identification flow chart is shown in Figure 1.

### *16 S rDNA sequencing*

The 16 S rDNA sequencing of mycobacterial DNA as the reference standard method for mycobacterial species identification was carried out using primer pair 8FPL (5’AGTTTGATCCTGGCTCAG 3’) and 1492 (5’GGTTACCTTGTTACGACT T 3’) as described by Turenne et al. [32]. The species were identified by comparing the 16 S rDNA sequences with similar sequences from GenBank.

## Abbreviations

PRA: Polymerase chain reaction restriction-enzyme analysis; DPRA: Duplex Polymerase chain Reaction restriction-enzyme Analysis; MTC: *Mycobacterium Tuberculosis* Complexes; NTM: Non-Tuberculous Mycobacteria; CE: Capillary Electrophoresis; AFB: Acid-Fast Bacillus; MAC: *M. Avium-intracellulare* Complex; LJ: Löwenstein-Jensen.

## Competing interest

The authors declare that they have no competing interests.

## Authors’ contributions

CCH wrote the manuscript. CSC, JHC, STH participated in the study design, and analysis. GHS and WCH managed the project. JYH, JJL assisted in improving the manuscript. All authors read and approved the final manuscript.
